# Intraoperative neuromonitoring for preservation of the external branch of the superior laryngeal nerve in transoral endoscopic thyroidectomy: initial experience from Vietnam

**DOI:** 10.3389/fsurg.2026.1870052

**Published:** 2026-08-03

**Authors:** Hau Xuan Nguyen, Hien Xuan Nguyen, Nhat Tan Nguyen, Quang Van Le

**Affiliations:** 1 Department of Oncology, Hanoi Medical University, Hanoi, Vietnam; 2 Oncology Center, Hanoi Medical University Hospital, Hanoi, Vietnam; 3 Quan Su 2 Surgery Department, National Cancer Hospital, Hanoi, Vietnam

**Keywords:** external branch of the superior laryngeal nerve, intraoperative neuromonitoring, thyroid surgery, TOETVA, transoral endoscopic thyroidectomy

## Abstract

**Objectives:**

To evaluate the feasibility of intraoperative neuromonitoring (IONM) in identifying the external branch of the superior laryngeal nerve (EBSLN), classifying its anatomical variations, and to evaluate short-term vocal function in patients undergoing transoral endoscopic thyroidectomy vestibular approach (TOETVA).

**Methods:**

A prospective descriptive study was conducted on 30 patients with thyroid nodules who underwent TOETVA with IONM using the NIM 3.0 system (Medtronic) at Hanoi Medical University Hospital from September 2021 to September 2024. EBSLNs were identified using a standardized intermittent stimulation protocol (1 mA) during superior pole dissection and classified according to the Cernea system. Voice outcomes were assessed using the Voice Handicap Index-10 (VHI-10) and laryngoscopy preoperatively and at 3 months post-surgery.

**Results:**

A total of 31 EBSLNs were assessed (1 total thyroidectomy). IONM facilitated a 100% identification rate. The anatomical distribution according to Cernea classification was: Type 1: 11/31 (35.5%, 95% CI: 19.2–54.6%), Type 2a: 18/31 (58.1%, 95% CI: 39.1–75.5%), and Type 2b (high-risk variant): 2/31 (6.5%, 95% CI: 0.8–21.4%). No loss of EBSLN signal was recorded before the end of surgery. Mean VHI-10 scores showed no clinically significant change from baseline (2.1 ± 1.5) to 3 months postoperatively (3.4 ± 2.2; mean difference 1.3, 95% CI: −0.2 to 2.8, *p* = 0.08). Only 3/30 patients (10%) had VHI-10 increase >5 points (minimal clinically important difference). Transient recurrent laryngeal nerve palsy occurred in 1/30 (3.3%) and fully recovered within one month. No permanent nerve injuries were recorded.

**Conclusions:**

IONM-assisted EBSLN mapping during TOETVA is technically feasible and allows consistent intraoperative identification. In this preliminary series, no clinically apparent deterioration in voice function was observed. However, given the descriptive design and limited functional assessment tools, comparative studies incorporating objective acoustic measures are required to determine the incremental benefit of routine IONM use for EBSLN preservation.

## 1 Introduction

In thyroid surgery, while the recurrent laryngeal nerve (RLN) has long been the primary focus of intraoperative preservation, the external branch of the superior laryngeal nerve (EBSLN) remains frequently overlooked—earning it the designation of the “neglected nerve” (1). This small nerve (0.2–0.8 mm in diameter) innervates the cricothyroid muscle, which is responsible for tensing and lengthening the vocal cords during phonation. Injury to the EBSLN does not cause overt hoarseness but results in subtle yet functionally significant voice changes: vocal fatigue, reduced range, and loss of high-frequency phonation—consequences particularly devastating for professional voice users (2, 3).

The vulnerability of the EBSLN stems from its variable anatomical course in relation to the superior thyroid pole. The Cernea classification categorizes these variations into three types, with Type 2b (nerve crossing below the superior pole) representing the highest-risk variant, occurring in 6%–15% of patients (4). In thyroid surgery generally, visual identification rates of the EBSLN vary widely, ranging from 33% to 93% depending on surgeon experience and the use of adjunctive techniques (5). Without intraoperative neuromonitoring (IONM), the EBSLN can be difficult to identify even in open surgery, particularly in cases with large goiters or unfavorable anatomy (6).

In transoral endoscopic thyroidectomy vestibular approach (TOETVA), the craniocaudal trajectory offers a favorable perspective for visualizing the superior pole region. However, the restricted operative space and the steep angle of approach create specific technical challenges for EBSLN preservation (7, 8). Studies have shown that visual identification rates in TOETVA can be as low as 10% without IONM assistance, highlighting the need for adjunctive techniques in this minimally invasive approach (9).

Intraoperative neuromonitoring (IONM) has been shown to increase EBSLN identification rates to over 90% in international studies, yet its application in TOETVA remains limited, and no data exist for the Vietnamese population (10). This study presents the initial Vietnamese experience with intermittent IONM (NIM 3.0 system) during TOETVA, with the following objectives: to determine the identification rate and anatomical distribution of the EBSLN according to Cernea classification, and to evaluate initial postoperative voice outcomes and safety profile at 3-month follow-up.

## 2 Materials and methods

### 2.1 Study design and population

A prospective descriptive study was conducted on 30 patients with thyroid nodules who underwent transoral endoscopic thyroidectomy vestibular approach (TOETVA) with intraoperative neuromonitoring (IONM) at Hanoi Medical University Hospital, Vietnam, from September 2021 to September 2024.

Inclusion criteria: Patients aged 18–70 years with benign nodules ≤6 cm or differentiated thyroid carcinoma cT1N0M0 (≤2 cm), normal preoperative vocal cord mobility, and completed VHI-10 questionnaire.

Exclusion criteria: Preoperative recurrent laryngeal nerve (RLN) paralysis, history of neck surgery or radiation, contraindications to general anesthesia or transoral approach, pregnancy, and inability to complete follow-up.

### 2.2 Surgical technique and intraoperative neuromonitoring protocol

All procedures were performed by two experienced endocrine surgeons following the standardized TOETVA technique previously described by our group (11).

The NIM 3.0 system (Medtronic, Jacksonville, FL, USA) with specialized endotracheal tube was used for intermittent IONM. Tube placement was verified by impedance <5 k*Ω* bilaterally. Muscle relaxant (rocuronium 0.3 mg/kg) was used only for induction.

Stimulation parameters: 1 mA for EBSLN identification, 1–2 mA for RLN and vagus nerve; pulse width 100 *μ*s; frequency 4 Hz; positive response threshold >100 μV.

Standardized sequence: V1 (vagus stimulation before dissection), R1 (RLN identification), R2 (RLN after dissection), V2 (final vagus stimulation) following International Neural Monitoring Study Group guidelines (12). EBSLN mapping: Systematic stimulation of cricothyroid region during superior pole dissection. EBSLN confirmation: Final stimulation at end of superior pole dissection.

EBSLN identification: After sternothyroid muscle division, the cricothyroid region was systematically mapped from lateral to medial using a long curved Prass probe. Identified nerves were classified according to Cernea's classification (13):

Type 1: crosses >1 cm above superior thyroid pole.

Type 2a: crosses within 1 cm above superior thyroid pole.

Type 2b: crosses below superior thyroid pole (high-risk).

Superior pole vessels were ligated only after confirming negative stimulation in target tissue with ≥3 mm safety margin.

### 2.3 Data collection

Data were collected prospectively using standardized case report forms and entered into a secure electronic database. Variables included:

Demographic: Age, gender, occupation, voice demands.

Clinical: Nodule size, TIRADS classification, fine-needle aspiration results.

Surgical: Operative time, EBSLN identification time, Cernea type, IONM parameters (V1 amplitude, V2 amplitude, signal interference).

Follow-up: VHI-10 scores (14, 15), laryngoscopy findings, complications.

### 2.4 Statistical analysis

Data were analyzed using SPSS 27.0. Descriptive statistics presented as mean ± SD or *n* (%). Categorical variables reported with 95% confidence intervals (Wilson method). Preoperative and postoperative VHI-10 scores compared using paired t-test. *P* < 0.05 considered statistically significant.

### 2.5 Ethics

This study was approved by the Ethics Committee of Hanoi Medical University. All participating patients provided voluntary informed consent, and personal information was kept confidential.

## 3 Results

### 3.1 Clinical and pathological characteristics

A total of 30 patients were enrolled in this study, including 28 females (93.3%) and 2 males (6.7%), with a mean age of 39.7 ± 7.9 years (range: 24–61 years). One patient underwent total thyroidectomy, resulting in 31 EBSLNs at risk for assessment. The baseline characteristics are summarized in Table 1.

**Table 1:** Baseline characteristics of the study population (*n* = 30).

Characteristics	Value (mean ± SD/n, %)
Age (years)	39.7 ± 7.9
Gender, *n* (%)	
*Female*	28 (93.3)
*Male*	2 (6.7)
Indication for surgery, *n* (%)	
*Suspicious nodule (TIRADS 4-5)*	24 (80.0)
*Compressive symptoms*	4 (13.3)
Patient preference	2 (6.7)
Histopathology, *n* (%)	
*Malignant*	23 (76.7)
*Benign*	7 (23.3)
Tumor size on ultrasound (mm), mean ± SD	
*Malignant nodules*	7.2 ± 2.9
*Benign nodules*	28.9 ± 5.8
Type of surgery, *n* (%)	
*Lobectomy*	29 (96.7)
*Total thyroidectomy*	1 (3.3)

### 3.2 EBSLN identification and preservation

**Figure 1:**
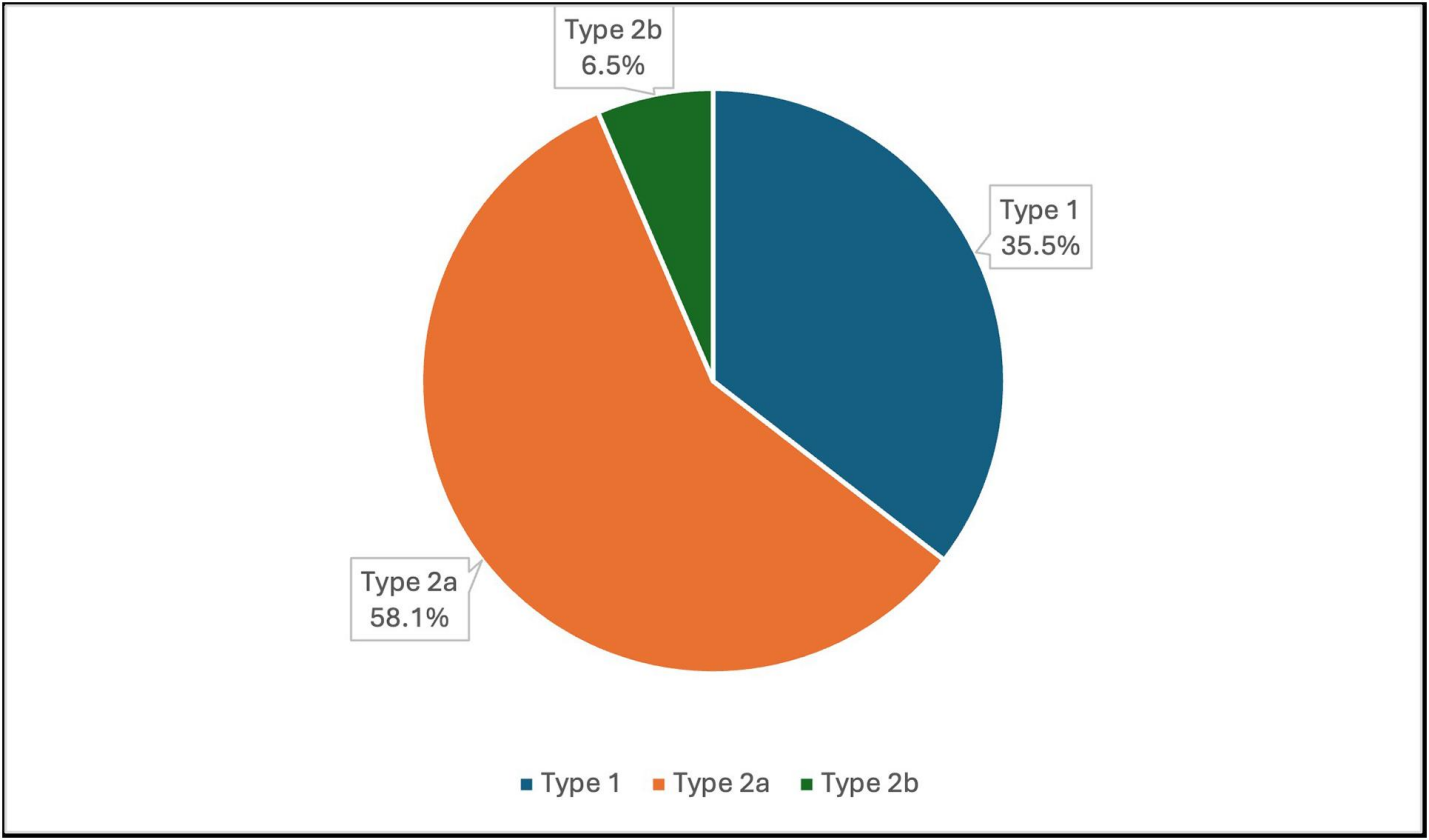
Distribution of EBSLN anatomical variations according to cernea's classification. Values are expressed as percentages for the anatomical variations of EBSLN according to Cernea's classification.

Intraoperative neuromonitoring enabled the successful identification of all 31 EBSLNs (100% identification rate). The anatomical distribution according to Cernea's classification is presented in Figure 1. Type 2a was the most frequent variant (58.1%), followed by Type 1 (35.5%). The high-risk Type 2b variant was identified in 2 nerves (6.5%).

### 3.3 Intraoperative neuromonitoring parameters

Intraoperative neuromonitoring parameters are summarized in Table 2. The mean V1 amplitude (initial vagus stimulation) was 642.5 ± 215.8 μV, and the mean V2 amplitude (final vagus stimulation) was 618.3 ± 208.4 μV, with no significant amplitude change (mean difference −24.2 μV, 95% CI: −58.3 to 9.9). The mean time required for EBSLN identification was 2.8 ± 1.2 min (range: 1.2–5.8 min). No loss of EBSLN signal was recorded before the end of surgery. Signal interference occurred in 14 patients (46.7%), primarily from energy devices (64.3% of interferences). All interference episodes were managed successfully without compromising nerve identification or surgical safety.

**Table 2:** Intraoperative neuromonitoring (NIM) parameters.

Parameter	Mean ± SD/n (%)	Range
Endotracheal tube placement time (min)	1.7 ± 1.1	0.8–4.5
Time to identify EBSLN (min)	2.8 ± 1.2	1.2–5.8
V1 amplitude (μV)	642.5 ± 215.8	312–1,156
V2 amplitude (μV)	618.3 ± 208.4	298–1,089
EBSLN amplitude at identification (μV)	327.6 ± 142.3	118–678
Signal interference rate, *n* (%)	14 (46.7)	
Interference from energy devices	9/14 (64.3)	
Interference requiring pause	6/30 (20.0)	

### 3.4 Voice outcomes

Voice outcomes assessed by VHI-10 are presented in Table 3. The mean total VHI-10 score showed no significant change from preoperative baseline (2.1 ± 1.5) to 3 months postoperatively (3.4 ± 2.2), with a mean difference of 1.3 (95% CI: −0.2 to 2.8, *p* = 0.08). No patient reported symptoms suggestive of EBSLN injury (vocal fatigue or loss of high-frequency phonation).

**Table 3:** Voice handicap Index-10 scores at baseline and 3 months postoperatively.

Parameter	Preoperative (*n* = 30)	3 months (*n* = 30)	Mean difference (95% CI)	*p*-value
Total VHI-10 score	2.1 ± 1.5	3.4 ± 2.2	1.3 (−0.2 to 2.8)	0.08

### 3.5 Postoperative complications

Postoperative complications are summarized in Table 4. No permanent nerve injuries, clinical EBSLN injury, or surgical site infections were recorded. Mental nerve injury presenting as chin paresthesia occurred in one patient (3.3%) and resolved within one months.

**Table 4:** Postoperative complications (*N* = 30).

Complications	n (%)	Recovery time
Transient RLN palsy	1 (3.3)	1 month
Permanent RLN palsy	0 (0)	-
High-pitch voice loss (EBSLN injury)	0 (0)	-
Transient hypocalcemia	0 (0)	-
Mental nerve injury (paraesthesia)	1 (3.3)	1 month
Subcutaneous emphysema	2 (6.7)	2–5 day

## 4 Discussion

This prospective study represents the first report from Vietnam on the use of intraoperative neuromonitoring for EBSLN preservation during TOETVA. Our findings demonstrate that intermittent IONM with the NIM 3.0 system enables reliable identification of the EBSLN in all cases, reveals the distribution of anatomical variants in a Vietnamese cohort, and contributes to preservation of vocal function with no permanent nerve injuries.

The advent of transoral endoscopic thyroidectomy vestibular approach (TOETVA) marks a significant advancement in meeting patients’ demand for “scarless” cosmetic outcomes (16–18). However, the core element and greatest challenge for surgeons remains the preservation of vital neural structures within a confined working space and a unique inferior-to-superior anatomical approach. While the recurrent laryngeal nerve (RLN) has long been a primary focus of intraoperative neuromonitoring, the external branch of the superior laryngeal nerve (EBSLN) is often considered the “forgotten nerve" (19). Due to its small size (approximately 0.2-0.8 mm in diameter) and anatomical variability, the EBSLN is frequently overlooked or difficult to identify during superior pole dissection. Injury to the EBSLN—the branch that innervates the cricothyroid muscle—typically does not cause overt hoarseness but instead leads to subtle yet significantly impactful voice impairments: vocal fatigue, loss of high pitch range, and alterations in the pitch and flexibility of speech and singing (2). These consequences are particularly severe for professional voice users.

The 100% EBSLN identification rate achieved in this study compares favorably with previous reports. Barczyński et al. reported an identification rate of 83.8% in thyroidectomy with IONM versus 34.3% without IONM (10), while Gurleyik et al. found visual identification rates as low as 30.4% in redo thyroidectomy without IONM (65.3% in primary cases) (20). In open thyroidectomy, visual identification rates vary widely, ranging from 33% to 93% depending on surgeon experience and adjunctive techniques (21). A national survey in Spain found that only 40% of surgeons routinely identify the EBSLN visually and merely 11% routinely stimulate it (22). This aligns with a 2016 INMSG survey showing that over 50% of surgeons never used IONM for the EBSLN due to inexperience, despite 93% agreeing it is essential for voice professionals. IONM use was significantly higher among high-volume (68.4%) versus low-volume surgeons (26.3%) (*P* = 0.004) (23). Our results confirm that systematic IONM mapping of the cricothyroid region overcomes the limitations of visual inspection alone, particularly in TOETVA where the steep approach angle hinders direct visualization of the superior pole (24). The mean time required for EBSLN identification was 2.8 ± 1.2 min, comparable to previous reports and demonstrating that IONM can be integrated into TOETVA without significantly prolonging operative time (24). This efficiency likely reflects the standardized mapping protocol and the use of a dedicated nerve probe.

The distribution of EBSLN anatomical variants according to the Cernea classification in our study (Type 1: 35.5%, Type 2a: 58.1%, Type 2b: 6.5%) is consistent with the international literature (25, 26). The Cernea classification is the most widely recognized system for assessing the risk of EBSLN injury based on its relationship with the superior thyroid pole and the superior thyroid artery (6). Clinical data indicate that Type 2 variants (2a and 2b) occur in approximately 65% of cases, emphasizing that the majority of patients have an EBSLN located within the danger zone during superior pole vessel ligation (4). Particularly in patients with large goiters (volume exceeding 50 mL), the rate of Type 2b EBSLN may increase up to 25-fold due to upward displacement of the superior thyroid pole, encroaching upon the nerve's pathway (27). A study on 242 nerves by Li et al. (2023) using IONM reported distributions of 41.0%, 45.3%, and 10.8% for Cernea Types 1, 2a, and 2b, respectively (25). A previous cadaveric anatomical analysis also reported high-risk variant rates ranging from 15% to 68%, confirming that a significant proportion of these nerves are at risk of injury during manipulation of the superior thyroid pole (28). Our study recorded 93.5% as Types 1-2a and 6.5% as Type 2b, consistent with the general trend in the Southeast Asian region. The lower rate of Type 2b in our study (6.5%) compared to some international reports may be attributable to the limited sample size but nonetheless confirms the existence of this high-risk group within the Vietnamese population.

Although the proportion of Type 2b is not high, this group represents the subset most vulnerable to injury if dissection relies solely on visual identification. Comparative studies have demonstrated that isolated EBSLN palsy often occurs when electrophysiological identification is omitted, proving that visual identification alone is insufficient in the endoscopic era. The use of IONM enables surgeons to precisely locate the nerve before superior pole vessel ligation, ensuring maximal safety for vocal function.

Voice outcomes in our study showed no clinically significant deterioration in VHI-10 scores at 3 months (2.1 ± 1.5 vs. 3.4 ± 2.2, *p* = 0.08). No cases of high-pitched voice loss (EBSLN injury) were recorded postoperatively. These findings confirm the efficacy of a preservation strategy combining IONM use with meticulous dissection of the Joll avascular space in maintaining long-term vocal function for patients. Our results align with the established literature that EBSLN injury is preventable through systematic IONM application. However, it should be noted that even with anatomically and functionally preserved nerves (confirmed by final EMG signals), patients may still experience transient voice changes during the first 1−3 weeks post-surgery. This phenomenon is illustrated in the study by Wu et al., where VHI-10 scores increased to 10.58 at one week postoperatively (compared to a baseline of 3.00), but nearly returned to baseline after three weeks (29). These temporary changes are generally attributed to postoperative edema, mechanical trauma to the sternothyroid muscle and perilaryngeal tissues, or mild fibrosis of the cricothyroid muscle.

The signal interference rate of 46.6% in our study is higher than that in open surgery (15−25%) and reflects the specific challenges of IONM in the endoscopic environment (30). Primary causes include electrode displacement within the confined working space characteristic of the transoral endoscopic approach, along with issues related to endotracheal tube positioning and the use of muscle relaxants. This demands particularly close coordination between the surgical and anesthesia teams throughout the procedure, from endotracheal intubation and maintenance of anesthesia to airway management. Across the three phases of the study, the interference rate decreased from 60% (first 10 cases) to 35% (last 10 cases), demonstrating the effectiveness of various strategies: coordinating with the anesthesia team to maintain stable anesthetic depth and avoid muscle relaxants, utilizing bipolar rather than monopolar devices when possible, pausing ultrasonic shear activation during stimulation, verifying electrode position when interference persisted, and proactively re-stimulating after each critical dissection step. Despite the considerable interference rate, the benefits of IONM in preventing EBSLN injury and preserving long-term voice quality fully justify the effort and technical requirements that this method entails.

A subject of ongoing debate in the literature is whether transoral endoscopic thyroidectomy (TOETVA) or traditional open surgery provides better conditions for EBSLN identification and preservation. Each approach has its own advantages and limitations, but evidence from our study together with recent reports suggests that TOETVA—particularly when combined with IONM—may yield superior outcomes. In open surgery, EBSLN preservation relies on anatomical landmarks standardized over decades. The Joll space and Reeves space are the key structures for accessing the nerve. Open surgeons often employ a “nerve-avoidance” technique by ligating vessels flush with the thyroid gland rather than directly identifying the EBSLN. However, electromyographic assessments have demonstrated that injury rates with this technique remain alarmingly high, ranging from 28% to 58%—figures that underscore that visual identification alone is insufficient (31). Conversely, TOETVA approaches the superior pole in an inferior-to-superior direction, creating a completely different perspective. The most important technical distinction is the division of the sternothyroid muscle at its insertion on the thyroid cartilage, recommended in approximately 75% of TOETVA cases—a rate significantly higher than in other approaches (8). This maneuver expands the Joll space, allowing lateral retraction of the muscle and clear exposure of the EBSLN within the thin membrane overlying the inferior pharyngeal constrictor muscle. Studies have confirmed that this muscle division does not cause long-term voice changes, although it may contribute to transient postoperative voice. The superior advantage of TOETVA stems from two factors: endoscopic magnification and the 30-degree viewing angle. The endoscope enables surgeons to better visualize the superior thyroid pole, providing a clear view of the underside of the vessels—precisely where Type 2b EBSLN variants typically course. This perspective is difficult to achieve in open surgery without extensive dissection.

Other procedure-related complications in our study were notably low. Transient recurrent laryngeal nerve palsy occurred in only 3.3% of cases, with complete recovery within one month. Mental nerve injury, manifesting as chin paresthesia—a complication specific to the transoral approach—was also observed in 3.3% of patients and resolved within one month. These low complication rates are consistent with previous reports and demonstrate the safety of TOETVA when performed under neuromonitoring guidance (16, 32, 33).

Several methodological considerations should be taken into account when interpreting the findings of this study. Foremost among these is the absence of a control group, which means we cannot confidently determine whether the high EBSLN identification rate and favourable voice outcomes are attributable to IONM itself, the inherent advantages of the endoscopic craniocaudal perspective, meticulous surgical technique, or a combination of these factors. While previous research has demonstrated that IONM improves EBSLN identification compared with visual inspection alone (9, 10), our descriptive study design does not allow us to establish the superiority of IONM over conventional dissection without neuromonitoring. In addition, the modest sample size of 30 patients and 31 nerves restricts the generalisability of our results and, more importantly, prevents meaningful subgroup analysis, which is particularly relevant for the high-risk Cernea Type 2b variant. Only two such nerves (6.5%) were identified in our cohort, meaning we are underpowered to reliably assess whether IONM confers additional protection in this subgroup, where the nerve is most vulnerable to injury during superior pole vessel ligation. Furthermore, while the VHI-10 is a well-validated and practical patient-reported outcome measure (14, 15), it has inherent limitations for detecting isolated EBSLN dysfunction. Unlike recurrent laryngeal nerve injury, EBSLN palsy typically does not cause hoarseness or vocal fold immobility but rather presents with subtle voice changes, such as reduced pitch range, vocal fatigue, and difficulty with high-frequency phonation (2, 3); These specific deficits may not be fully captured by the VHI-10, particularly in patients who do not perceive significant voice handicap despite objective functional impairment. Moreover, normal laryngoscopy findings cannot exclude EBSLN injury because vocal cord mobility remains intact. Therefore, the stable VHI-10 scores in our series should not be interpreted as definitive evidence against subclinical nerve dysfunction. A further consideration is the relatively high signal interference rate of 46.6%, which exceeded that typically reported in open thyroidectomy series (15%–25%) and reflects the specific technical challenges of the endoscopic environment, including the confined working space and electrode displacement. Although this rate improved with experience, declining from 60% in the first ten cases to 35% in the last ten cases, it underscores the substantial learning curve associated with this technique and highlights the need for close coordination between surgical and anaesthesia teams. Taken together, these limitations indicate that our findings are preliminary and hypothesis-generating, and they require confirmation in larger comparative studies with objective voice assessments and longer follow-up before routine IONM use for EBSLN preservation can be recommended.

In the future, we will focus on several research directions, including in-depth anatomical studies of the EBSLN in both open and endoscopic surgery with larger patient cohorts to more precisely determine the rate of anatomical variants in the Vietnamese population, particularly Type 2b, and their correlation with factors such as nodule size, gender, and age. Randomized comparative studies between IONM and visual dissection alone in TOETVA are needed to quantify the added value of neuromonitoring. Long-term follow-up with objective voice assessment (acoustic analysis, maximum phonation time, laryngeal electromyography) to detect subclinical changes, cost-effectiveness analysis, and learning curve evaluation will support resource allocation decisions and the development of training programs. Furthermore, the development of emerging technologies such as continuous IONM (C-IONM), transcutaneous electrodes, or trocar-integrated electrodes is under investigation, holding promise for reducing the complexity of vagus nerve dissection in TOETVA. The integration of artificial intelligence for EMG waveform analysis may also usher in a new era of personalized neuromonitoring, enabling more accurate prediction of safe injury thresholds tailored to individual patients based on their unique anatomical and neurophysiological characteristics.

## 5 Conclusion

This preliminary study demonstrates that IONM-assisted EBSLN mapping during TOETVA is technically feasible and enables consistent nerve identification in a Vietnamese cohort. The anatomical distribution of Cernea variants in our series was broadly consistent with international reports. No permanent nerve injuries or clinically significant voice deterioration were observed at three-month follow-up. However, given the descriptive design, modest sample size, and limited number of high-risk Type 2b nerves, these findings should be interpreted with caution. These findings should be considered preliminary and require validation in future studies. The additional benefit of routine IONM over visual identification alone for EBSLN preservation in TOETVA remains to be established in comparative studies with objective voice assessments and longer follow-up.

## Data Availability

The raw data supporting the conclusions of this article will be made available by the authors, without undue reservation.
